# New Test for Prussic Acid

**Published:** 1846-08

**Authors:** 


*New Test for Prussic Acid.*—The following new method of testing for hydrocyamic acid is proposed by Mr. Richard Austin, Jr., of this city. The precipitate of cyanide of silver, say half a grain, obtained in the usual manner, is mixed with a small quantity of oxide of iron and carbonate of potash, and the whole fused together in an iron or platinum capsule. The fused mass is then dissolved in half an ounce of distilled water, filtered, and rendered slightly acid by the addition of a few drops of hydrochloric acid. The liquid thus treated is next divided into two portions, to one of which a few drops of a solution of sulphate of copper is added, which immediately causes the evolution of the chocolate-brown color, so characteristic of the ferro-cyanide of copper; and to the other a few drops of the muriate tincture of iron, or any persalt of iron, when the solution becomes intensely blue by the formation of the ferro-cyanide of iron, the ordinary Prussian blue.

In Mr. Austin's opinion, "these two tests, with the well-known odor of prussic acid, are, *independently of all others*, sufficient to convince the medical jurist of the presence of free prussic acid." Mr. Austin adduces several arguments to shew the superiority of this test to those already known to chemists, both in accuracy and facility of application, by persons not skilled in chemical manipulation. The precipitates above mentioned are very distinctly obtained with half a grain of cyanide of silver.—*Ibid.*—*Abridged from the Dublin Hospital Gazette.*

